# MicroRNA-203 predicts human survival after resection of colorectal liver metastasis

**DOI:** 10.18632/oncotarget.13816

**Published:** 2016-12-07

**Authors:** T. Peter Kingham, Hoang C.B. Nguyen, Jian Zheng, Ioannis T. Konstantinidis, Eran Sadot, Jinru Shia, Deborah Kuk, Steven Zhang, Leonard Saltz, Michael I. D’Angelica, William R. Jarnagin, Hani Goodarzi, Sohail F. Tavazoie

**Affiliations:** ^1^ Laboratory of Systems Cancer Biology, The Rockefeller University, New York, NY, USA; ^2^ Department of Surgery, Memorial Sloan Kettering Cancer Center, New York, NY, USA; ^3^ Department of Pathology, Memorial Sloan Kettering Cancer Center, New York, NY, USA; ^4^ Department of Epidemiology and Biostatistics, Memorial Sloan Kettering Cancer Center, New York, NY, USA; ^5^ Department of Medicine, Memorial Sloan Kettering Cancer Center, New York, NY, USA; ^6^ Department of Biochemistry and Biophysics, University of California, San Francisco, San Francisco, CA, USA; ^7^ Department of Urology, University of California, San Francisco, San Francisco, CA, USA; ^8^ Helen Diller Family Comprehensive Cancer Center, University of California, San Francisco, San Francisco, CA, USA

**Keywords:** microRNA, miR-203, colorectal liver metastasis, survival, resection

## Abstract

**Background:**

Resection of colorectal liver metastasis (CRLM) can be curative. Predicting which patients may benefit from resection, however, remains challenging. Some microRNAs (miRNAs) become deregulated in cancers and contribute to cancer progression. We hypothesized that miRNA expression can serve as a prognostic marker of survival after CRLM resection.

**Results:**

MiR-203 was significantly overexpressed in tumors of short-term survivors compared to long-term survivors. R1/R2 margin status and high clinical risk score (CRS) were also significantly associated with short-term survival (both *p* = 0.001). After adjusting for these variables, higher miR-203 expression remained an independent predictor of shorter survival (*p* = 0.010). In the serum cohort, high CRS and KRAS mutation were significantly associated with short-term survival (*p* = 0.005 and *p* = 0.026, respectively). After adjusting for CRS and KRAS status, short-term survivors were found to have significantly higher miR-203 levels (*p* = 0.016 and *p* = 0.033, respectively).

**Materials and Methods:**

We employed next-generation sequencing of small-RNAs to profile miRNAs in solid tumors obtained from 38 patients who underwent hepatectomy for CRLM. To validate, quantitative reverse-transcription polymerase chain reaction (qRT-PCR) was performed on 91 tumor samples and 46 preoperative serum samples.

**Conclusions:**

After CRLM resection, short-term survivors exhibited significantly higher miR-203 levels relative to long-term survivors. MiR-203 may serve as a prognostic biomarker and its prognostic capacity warrants further investigation.

## INTRODUCTION

Colorectal cancer (CRC) is the third most prevalent cancer in both men and women in the U.S., and about half of all recurrences after CRC resection involve the liver [1, 2]. Resection of colorectal liver metastasis (CRLM) offers a chance of cure in 1 in 5 patients [3]. The challenge is determining which patients are likely to recur early and die of their disease or to have a long disease-free interval or be cured. A prior study from Memorial Sloan Kettering Cancer Center (MSKCC) showed that median survival after resection for CRLM was roughly 42 months among 1001 consecutive patients [4]. A clinical risk score (CRS) from 0–5 that predicted outcome after CRLM resection was created, in which 1 point was given for each of 5 clinical variables [4]. These variables were composed of node-positive primary, disease free interval from CRC resection to CRLM diagnosis of < 12 months, number of CRLM > 1, largest CRLM size > 5 cm, and carcinoembryonic antigen (CEA) > 200 ng/mL [4]. CRS of 0 predicted median survival of 74 months, whereas a CRS of 5 predicted median survival of only 22 months [4]. On subsequent analysis, however, the CRS loses its significance after patients survive for several years [5].

As the CRS demonstrates, identifying consistent and reliable predictors of outcome after CRLM resection remains a universal challenge. The ability of most models using clinicopathological data to predict outcome leads to concordance indices of approximately 0.6, which is not much better than chance alone [6]. Because of these deficiencies, scoring systems do not typically influence the treatment options provided.

In recent years, investigators have also studied the impact of BRAF or KRAS mutational status on patient outcomes after CRLM resection. Approximately 25% of CRLM resected patients were found to harbor the KRAS mutations at codon 12 and 13 and 2–4% were found to harbor the BRAF V600E mutation [7–12]. Patients harboring these mutations experienced worse survival after resection, potentially due to driving of drug resistance by such mutations [8–12]. However, our surgical experience revealed that many patients with wild type KRAS and BRAF also exhibited short-term survival after CRLM resection, despite lacking these unfavorable mutations. Identifying an additional reliable prognostic biomarker could optimize patient selection for liver resection with adjuvant regional and/or systemic chemotherapy and help determine the course of follow-up surveillance for CRLM recurrence.

MicroRNAs (miRNA) are 19–25 nucleotide long post-transcriptional regulatory small non-coding RNAs which specifically downregulate target messenger RNA abundance and translation by binding to complementary sequences in the 3′ untranslated regions of transcripts [13]. Because of their critical roles in post-transcriptional regulation, aberrant expression of many miRNAs have been identified as critical in the multistep processes of cancer progression and metastasis either through their oncogenic or tumor suppressive functions [14–16]. CRC and CRLM have been associated with dysregulated expression of several miRNAs such as miR-145, miR-221, miR-18a, and miR-27a [17–24]. Despite the growing knowledge of the prognostic potential of miRNAs as biomarkers in cancers in general and of their importance in regulating CRC and CRLM in particular, there is still a paucity of validated miRNAs that predict survival outcome in cancers [25–29]. The aim of this study was to profile differentially expressed miRNAs in tumors and preoperative sera in patients with short-term versus long-term survival after CRLM resection.

## RESULTS

### Available clinicopathological variables provide inconclusive prognostic power

A total of 91 patients in the tumor cohort and 46 patients in the serum cohort who underwent initial CRLM resection were included in this study. In the tumor cohort, short-term survivors had a median overall survival of 8.4 months. Long-term survivors had a median overall survival of 21 years with median follow-up of 16 years. In the serum cohort, short-term survivors had a median overall survival of 15 months. Long-term survivors had a median overall survival that has not yet been reached with a median follow-up of 5 years. Patient demographic and tumor characteristics for tumor and serum cohorts are shown in Table 1A and 1B, respectively.

**Table 1A:** Patient demographic and tumor characteristics in the tumor cohort

	Total (*n* = 91)	Short-Term Survivors (*n* = 46)	Long-Term Survivors (*n* = 45)	*p*-value
Age	59 (28–83)	59 (28–83)	59 (28–79)	0.902
Male	57 (63)	30 (65)	27 (60)	0.668
CRC Location				
Colon	66 (73)	31 (67)	35 (78)	0.349
Rectum	25 (27)	15 (33)	10 (22)	
CRC Nodes				
Negative	42 (46)	17 (37)	25 (56)	0.094
Positive	49 (54)	29 (63)	20 (44)	
CRC TNM Stage at Presentation				
I	5 (5)	3 (7)	2 (4)	**0.008**
II	27 (30)	9 (20)	18 (40)	
III	31 (34)	13 (28)	18 (40)	
IV	28 (31)	21 (46)	7 (16)	
CEA				
< 200 ng/mL	76 (84)	37 (80)	39 (87)	0.483
≥ 200 ng/mL	9 (10)	6 (13)	3 (7)	
Disease Free Interval				
< 12 months	44 (48)	29 (63)	15 (33)	**0.006**
≥ 12 months	47 (52)	17 (37)	30 (67)	
CRLM Diagnosis				
Synchronous	28 (31)	21 (46)	7 (16)	**0.003**
Metachronous	63 (69)	25 (54)	38 (84)	
CRLM Largest Size				
< 5 cm	52 (57)	19 (41)	33 (73)	**0.003**
≥ 5 cm	39 (43)	27 (59)	12 (27)	
CRLM Numbers				
Single	39 (43)	17 (37)	22 (49)	0.293
Multiple	52 (57)	29 (63)	23 (51)	
CRLM Differentiation				
Moderate	57 (63)	34 (74)	23 (51)	0.275
Poor	8 (9)	3 (7)	5 (11)	
CRLM Resectability				
R0	70 (77)	27 (59)	43 (96)	**< 0.001**
R1	11 (12)	9 (20)	2 (4)	
R2	10 (11)	10 (22)	0 (0)	
Clinical Risk Score				
0–2 Low Risk	61 (76)	22 (48)	39 (87)	**< 0.001**
3–5 High Risk	30 (33)	24 (52)	6 (13)	
Chemotherapy				
Pre-CRLM Resection	53 (58)	33 (72)	20 (44)	**0.011**
Post-CRLM Resection				
Systemic only	31 (34)	18 (39)	13 (29)	0.585
Systemic + HAIP	27 (30)	12 (26)	15 (33)	
KRAS				
Mutant	10 (11)	8 (17)	2 (4)	0.170
Wild type	10 (11)	4 (9)	6 (13)	
BRAF V600E				
Mutant	0 (0)	0 (0)	0 (0)	−
Wild type	18 (20)	12 (26)	6 (13)	

Abbreviations: CRC, colorectal cancer; CRLM, colorectal liver metastasis; TNM, tumor node metastasis, CEA, carcinoembryonic antigen. HAIP, hepatic artery infusion pump.

**Table 1B:** Patient demographic and tumor characteristics in the serum cohort

	Total (*n* = 46)	Short-Term Survivors (*n* = 25)	Long-Term Survivors (*n* = 21)	*p*-value
Age	56 (26–78)	56 (26–78)	56 (36–73)	0.834
Male	31 (67)	17 (68)	14 (67)	1.000
CRC Location				
Colon	39 (85)	22 (88)	17 (81)	0.686
Rectum	7 (15)	3 (12)	4 (19)	
CRC Nodes				
Negative	14 (30)	5 (20)	9 (43)	0.117
Positive	32 (70)	20 (80)	12 (57)	
CRC TNM Stage at Presentation				
I	6 (13)	0 (0)	6 (29)	**0.009**
II	2 (4)	1 (4)	1 (5)	
III	10 (22)	5 (20)	5 (24)	
IV	28 (61)	19 (76)	9 (43)	
CEA				
< 200 ng/mL	40 (87)	20 (80)	20 (95)	0.114
≥ 200 ng/mL	4 (9)	4 (16)	0 (0)	
Disease Free Interval				
< 12 months	36 (78)	21 (84)	15 (71)	0.475
≥ 12 months	10 (22)	4 (16)	6 (29)	
CRLM Diagnosis				
Synchronous	27 (59)	19 (76)	8 (38)	**0.016**
Metachronous	19 (41)	6 (24)	13 (62)	
CRLM Largest Size				
< 5 cm	32 (70)	15 (60)	17 (81)	0.199
≥ 5 cm	14 (30)	10 (40)	4 (19)	
CRLM Numbers				
Single	14 (30)	5 (20)	9 (43)	0.117
Multiple	32 (70)	20 (80)	12 (57)	
CRLM Differentiation				
Moderate	37 (80)	18 (72)	19 (90)	0.111
Poor	4 (9)	4 (16)	0 (0)	
CRLM Resectability				
R0	29 (63)	13 (52)	16 (76)	0.236
R1	6 (13)	4 (16)	2 (10)	
R2	11 (24)	8 (32)	3 (14)	
Clinical Risk Score				
0–2 Low Risk	18 (39)	5 (20)	13 (62)	**0.006**
3–5 High Risk	28 (61)	20 (80)	8 (38)	
Chemotherapy				
Pre-CRLM Resection	36 (78)	25 (100)	11 (52)	**< 0.001**
Post-CRLM Resection				
Systemic only	18 (39)	8 (32)	10 (48)	0.423
Systemic + HAIP	26 (57)	15 (60)	11 (52)	
KRAS				
Mutant	15 (33)	12 (48)	3 (14)	**0.037**
Wild Type	28 (61)	12 (48)	16 (76)	
BRAF V600E				
Mutant	0 (0)	0 (0)	0 (0)	−
Wild type	31 (67)	16 (64)	15 (71)	

Abbreviations: CRC, colorectal cancer; CRLM, colorectal liver metastasis; TNM, tumor node metastasis, CEA, carcinoembryonic antigen. HAIP, hepatic artery infusion pump.

In the tumor cohort, CRC tumor node metastasis (TNM) stage, disease free interval (DFI) between CRC resection and CRLM diagnosis, largest CRLM tumor, CRLM margin status, and CRS were significantly different between short-term versus long-term survivors. When the CRS was stratified as low versus high risk, there was a significant difference between the two cohorts. However, of the 46 patients that were considered short-term survivors in the tumor cohort, 22 patients (48%) had a low risk CRS. Similarly, only 39 of 61 patients (64%) with a low CRS were long-term survivors.

In the serum cohort, patients’ CRC TNM stage, CRLM synchronous or metachronous diagnosis, CRS, and KRAS mutation status were significantly different between short-term and long-term survivors. 48% of short-term survivors in the serum cohort harbored the KRAS mutation, compared to only 14% of the long-term survivors, which was significantly different (*p* = 0.037). Among these 15 KRAS mutants, 6 patients had mutations at G12D, 3 at G13D, 3 at G13V, 2 at G12C, and 1 at G13C. None of the 31 patients in the serum cohort tested for BRAF had a V600E mutation.

### Higher tumor miR-203 expression is associated with worse post-resection survival

**Figure 1:**
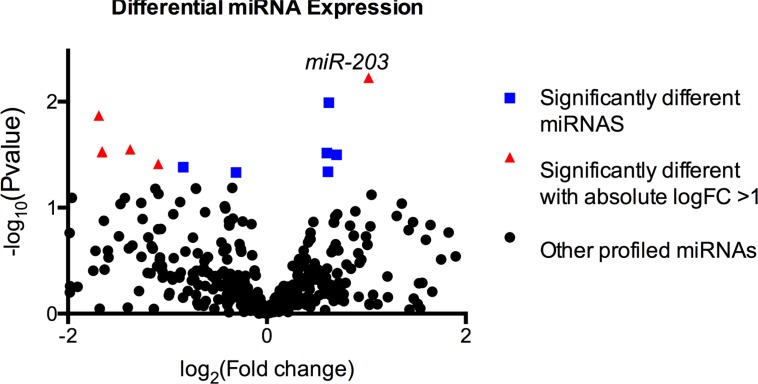
Next-generation sequencing of small-RNAs showed 16 miRNAs (squares and triangles) that were significantly differentially expressed, of which 10 miRNAs (triangles) exhibited an absolute fold change of 2 or above MiR-203 expression levels were significantly different between short-term vs. long-term survivors (log_2_Foldchange = 1.02, *p* = 0.006).

**Figure 2:**
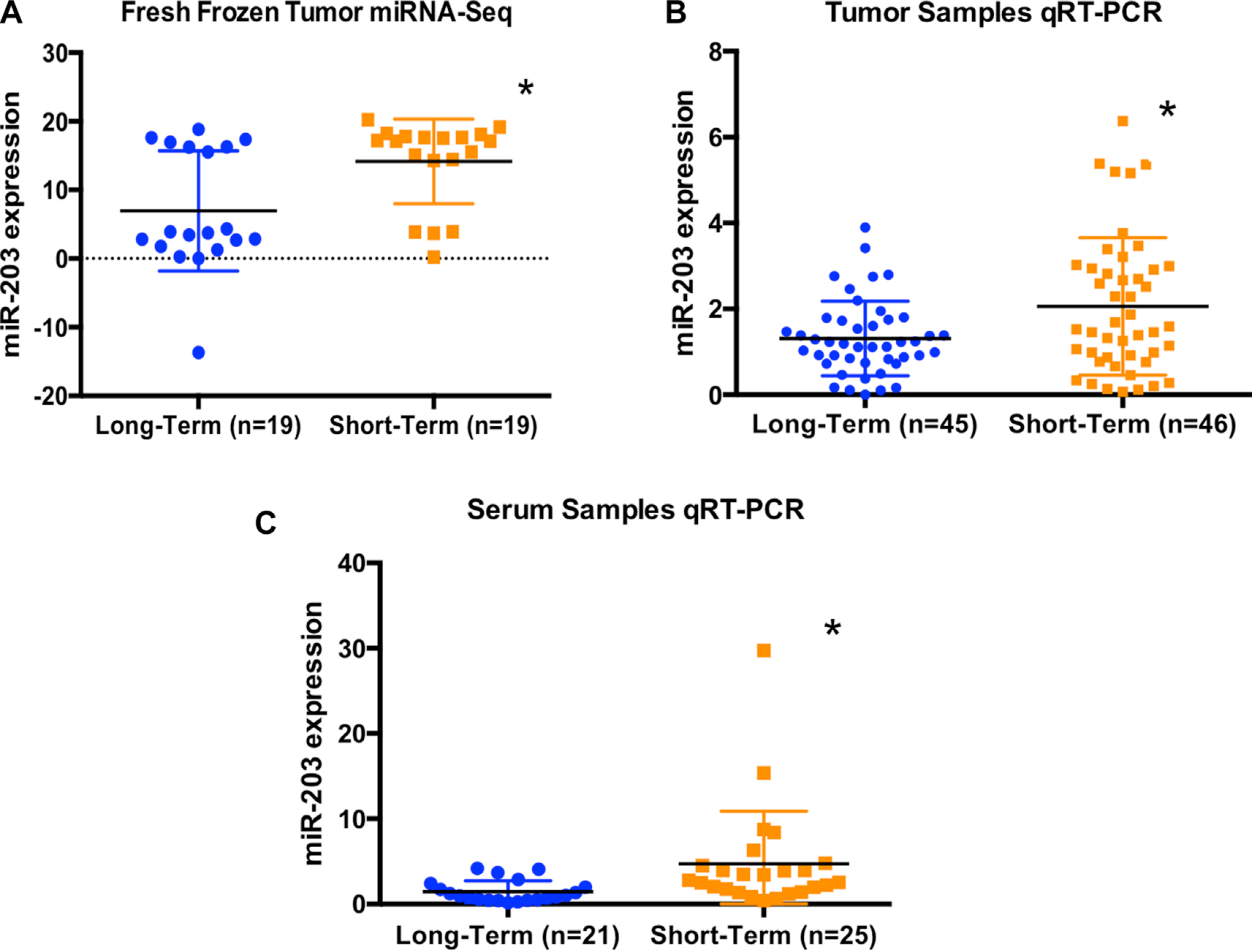
(**A**) Absolute expression level of miR-203 from next-generation sequencing analysis of 38 fresh frozen tumors demonstrated a significant upregulation of miR-203 among short-term survivors (*p* = 0.006). (**B**) Relative expression level of miR-203 from qRT-PCR of 91 tumor samples showed that short-term survivors exhibited significantly higher expression of miRNA-203 compared to long-term survivors (*p* = 0.020, one-sided Wilcoxon rank sum test). (**C**) Relative expression level of miR-203 from qRT-PCR of 46 serum samples showed that short-term survivors exhibited significantly higher expression of miRNA-203 compared to long-term survivors (*p* < 0.001).

Through unbiased small-RNA sequencing of human tumor RNAs, we identified miR-203 as a miRNA that is differentially expressed between the two survivor groups studied (Figure 1, log_2_Foldchange = 1.02, *p*-value = 0.006). Expression level of tumoral miR-203 was significantly increased among short-term survivors (Figure 2A, *p* = 0.006). To validate this finding, we re-measured miR-203 in this group of 33 patients plus an additional cohort of 58 patients using qRT-PCR. In this expanded cohort of 91 patients, we observed a significantly higher miR203 expression in patients with poor prognosis (Figure 2B, *p* = 0.020, one-sided Wilcoxon rank sum test).

### Elevated serum miR-203 levels is associated with worse post-resection survival

To assess the potential of miR-203 as a minimally invasive biomarker, we obtained preoperative sera from an independent cohort of 46 patients with CRLM. Preoperative sera of short-term survivors contained significantly higher levels of miRNA-203 by qRT-PCR compared to long-term survivors (Figure 2C, *p* < 0.001).

### MiR-203 expression can be used as an independent prognostic biomarker

In univariate analyses as shown in Table 2A, lower miR-203 expression in CRLM tumor samples, as well as negative margin and low risk CRS were all significantly associated with long-term survival (*p* = 0.012, *p* = 0.001, *p* = < 0.001, respectively). After adjusting for margin status and CRS, lower expression of miR-203 remained significantly associated with long-term survival (OR = 0.52, *p* = 0.010). Moreover, the addition of information gained from miR-203 expression improved the concordance index, which was 0.776 based on margin status and CRS, to 0.843.

**Table 2A:** Tumor cohort logistic regression was performed with long-term survivorship as the outcome

	Univariate Analysis				Multivariate Analysis			
	OR	95% Lower	95% Upper	*p*-value	OR	95% Lower	95% Upper	*p*-value
miRNA	0.617	0.424	0.897	**0.012**	0.517	0.314	0.851	**0.010**
Margin (R1/R2 vs R0)	0.066	0.014	0.307	**0.001**	0.066	0.013	0.344	**0.001**
Clinical risk score (high vs low)	0.141	0.050	0.397	**< 0.001**	0.169	0.052	0.555	**0.003**

**Table 2B:** Serum cohort logistic regression was performed with long-term survivorship as the outcome

	Univariate Analysis				Multivariate Analysis			
	OR	95% Lower	95% Upper	*p*-value	OR	95% Lower	95% Upper	*p*-value
miRNA	0.527	0.324	0.859	**0.010**	0.506	0.292	0.879	**0.016**
Clinical risk score (high vs low)	0.154	0.041	0.575	**0.005**	0.102	0.019	0.537	**0.007**

**Table 2C:** Serum cohort logistic regression was performed with long-term survivorship as the outcome

	Univariate Analysis				Multivariate Analysis			
	OR	95% Lower	95% Upper	*p*-value	OR	95% Lower	95% Upper	*p*-value
miRNA	0.527	0.324	0.859	**0.010**	0.568	0.338	0.954	**0.033**
KRAS (mutant vs wild type	0.188	0.043	0.815	**0.026**	0.177	0.035	0.883	**0.035**

Similarly, in the CRLM serum cohort, univariate analysis showed that low risk CRS and wild type KRAS were significantly associated with long-term survival (Table 2B and 2C, p = 0.005 and *p* = 0.026, respectively). After adjusting for CRS and KRAS status, miRNA-203 differential expression in long-term survivors remained significant (Table 2B and 2C, p = 0.016 and *p* = 0.033, respectively).

## DISCUSSIONS

In this study, we identified miR-203 to be differentially regulated between short and long-term survivors after CRLM resection. This is important because prognostic markers can help optimize treatment plans. For example, patients with a small burden of disease but who are deemed high risk according to a prognostic marker may be offered chemotherapy trials instead of resection. This can allow for time to observe the progression of disease and to assess tumor chemosensitivity. The reverse is also true. A patient with small volume extrahepatic disease but who is deemed low risk may be offered a resection, which is usually not the standard treatment approach.

The power of clinical risk scores is that they rely on data that is available prior to surgery and do not rely on factors such as response to treatment or comprehensive pathologic evaluation. These scores have been used to predict outcomes for patients with CRLM. While often significant in predicting disease-specific survival in single institution studies, some external validation attempts have failed to duplicate these findings [30]. This has led investigators to search for additional prognostic factors. Preoperative serum levels of angiogenesis factors, for example, such as placenta growth factor (PIGF), combined with circulating tumor cell levels, have been identified as independent predictors of poor recurrence-free survival in a series of 107 patients [31]. There are also molecular risk scores that have been applied to patients with CRLM. Three separate tumor gene signatures have been developed at MSKCC to predict disease specific survival (19 genes), hepatic recurrence-free survival (115 genes), and overall survival (20 genes) [32, 33]. Up to 6 genes overlap among these gene signatures [32, 33]. The challenge in applying these molecular risk scores is that they require pathologic evaluation, which may require additional biopsies and testing of multiple genes, and often are comprised of genes that are not critical in metastasis. Given these limitations, this study was initiated to find miRNA as CRLM biomarkers.

Prognostic biomarkers reflect the intricate underlying biology that enables cancer cells to progress and cause worse patient outcome. Thus, the search for prognostic biomarkers parallels with much of the recent efforts to study metastatic mechanisms. The differential miRNA expression landscape among patients with varying stages of CRC including CRLM suggests that miRNA dysregulation is one of the mechanisms underlying multi-step metastatic progression. For instance, our group recently showed that miRNA 483-5p and 551a were downregulated in metastatic CRC [15]. It was shown that both of these miRNAs shared a surprising metabolic target, CKB (Creatinine Kinase, Brain-type), which creates a reservoir of phosphocreatine for ATP generation that permits CRC cell survival during hepatic colonization where hypoxic stress impairs outgrowth [15]. In a study by another group, miRNA-99b-5p was shown to be expressed at higher levels in metastasis-free CRC patients relative to tumors of those who developed CRLM [25]. These studies suggested that deregulated miRNA expression both associates with and drives CRC progression.

The clinical importance of miR-203 in colorectal cancer has been poorly characterized until recently. MiR-203 was found to be significantly higher in plasma of stage IV CRC patients compared to earlier staged CRC [27]. Another group also observed higher serum levels of miR-203 in CRLM compared to matched CRC, and showed that increased levels were associated with worse survival and metastasis to liver as well as lymph nodes, peritoneum, and distant organs [28]. This growing body of evidence implicated the important role of miR-203 level in regulating CRC progression. Our unbiased study also implicated miR-203 in CRC pathogenesis. Specifically, this was the first study showing miR-203 differential expression to be associated with survivorship after CRLM resection. Importantly, the concordance index for the CRS and margin status was higher when miR-203 expression level was added. It is possible that miR-203, after study in a larger cohort, will enhance our ability to personalize treatment plans by capturing the appropriate level of risk associated with a patient's CRLM.

Relative expressions and biological roles of miRNA-203 in CRC and CRLM remain to be elucidated. MiR-203 expression was shown to be upregulated in both CRC and CRLM compared to normal colon mucosa and implicated in epithelial to mesenchymal transition [34]. Another group reported that miRNA-203 was observed to be downregulated in both CRC and CRC cell lines compared to normal colon mucosa, and that lower expression was associated with larger CRC tumor and more advanced stage tumors [35]. Even though potential downstream targets of miR-203 were not included in the scope of this study, there have been several reports that suggest a wide scope of potential candidate targets whose levels are modulated by miR-203. One report implicated miR-203 as a negative regulator of ZNF 217 [36]. In lung cancer cells, miR-203 was found to promote apoptosis through targeting SRC [37]. PIK3CA was also shown to be a direct target of miR-203, whose downregulation was found to increase AKT signaling in gastric cancer [38]. MiR-203 is also predicted to target the 3′UTR of several genes for which aberrant expression levels have been associated with tumorigenesis and metastasis, such as PTP4A, PRKCB, and RAB10 [39]. These studies in aggregate suggest both cancer promoting and suppressive potential for miR-203 in CRC depending on the context. Our findings support a role for miR-203 in promoting colorectal cancer metastatic relapse. Needless to say, the causal role of miR-203 in the initiation, promotion, and relapse of CRLM remain to be investigated.

In addition to CRC and CRLM, miRNA-203 expression was found to be deregulated in various cancer types, which may potentially reflect the tissue specific nature of miRNA-203. MiR-203 was shown to be down-regulated in melanoma [40], hepatocellular carcinoma [41], and cholangiocarcinoma [42]. On the other hand, miRNA-203 was overexpressed in pancreatic adenocarcinoma [43], prostate cancer [44], ovarian cancers [45], and metastatic breast cancer [46].

In conclusion, short-term survivors after CRLM resection exhibit significantly higher miR-203 expression in their CRLM tissues and preoperative sera compared to long-term survivors. Curative intent of surgical resection can be further strengthened by prognostic factors including CRS, mutational status, and miR-203 expression. The prospect of knowing in advance the likelihood of survival in patients after CRLM surgery provides physicians with invaluable information for counseling patients and developing an optimal treatment plan. These studies warrant further large-scale studies to develop an approach to stratify patients according to their miR-203 status and additional molecular factors.

## MATERIALS AND METHODS

### Study population

With approval of the Institutional Review Board at MSKCC, specimens from 150 patients who underwent liver resection for CRLM from 1990 to 2014 were collected. Samples from 100 patients were used in the tumor cohort, and samples from an additional 50 patients were used in the serum cohort.

In the tumor cohort, 50 short-term survivors and 50 long-term survivors were selected. Short-term survivors were defined in the tumor cohort as patients who died of metastatic disease within 2.5 years after resection, whereas long-term survivors were patients who survived 5 years beyond resection. These time points were selected to allow for adequate sample size and because patients with unresectable liver metastases treated with modern chemotherapy regimens survive on average between 2 and 2.5 years [47, 48]. Five patients were excluded due to poor tissue miRNA quality and 4 patients were excluded for having had prior hepatectomy for CRLM. Thus, final analysis was based on 91 patients whose tumoral miRNAs were extracted after first occurrence of CRLM. We performed next-generation small-RNA sequencing on fresh frozen tumors from 38 patients. To validate the findings from the next-generation, qRT-PCR was performed on 33 of the 38 original patients for whom we had adequate fresh frozen tumors plus an additional 58 patients for whom we had available formalin-fixed, paraffin embedded (FFPE) tissue slides for RNA extraction. In total, the tumor cohort consisted of 91 patients whose tumoral miRNA expression levels were quantified and normalized by qRT-PCR.

In the serum cohort, 25 short-term survivors and 25 long-term survivors were selected with preoperative sera obtained within 5 weeks of CRLM resection (median of 7 days; range 0–33 days). Four patients were excluded due to poor sample miRNA quality. Thus, the final analysis was based on 46 patients whose serum miRNAs were extracted and quantified by qRT-PCR. A new group of patients were selected for the serum cohort because most patients in the tumor cohort lacked preoperative serum collection for research.

Patient information including demographic and relevant clinicopathological variables were obtained from a retrospective review. Preoperative laboratory results as well as details of the primary CRC and CRLM resection and pathology, KRAS and BRAF mutation status, subsequent recurrence, last follow up, and death were recorded. Clinical risk scores (CRS) were calculated for each patient as previously described by Fong et al, and it was further stratified as low risk (0–2) versus high risk (3–5) [4].

### RNA extraction

From MSKCC's prospectively maintained tissue bank, fresh frozen tumors or formalin fixed, paraffin embedded (FFPE) tissues mounted on glass slides were obtained for each patient. Solid tumor samples were flash frozen, preserved in RNAlater (Thermofisher AM7024) to minimize RNA degradation, cut and measured to roughly the same size, and grounded with disposable pestles (Sigma Z359947) before being subjected to RNA extraction using the Norgen miRNA purification kit (21300) per manufacturer's instructions. Extracted RNA was then bioanalyzed for integrity prior to library preparation. To extract RNA from FFPE tissue slides, a pathologist (J.S.) reviewed the H&E slides for visual confirmation of tumor cells, and areas containing tumor cells were encircled for extraction. Tumor cells were scraped from corresponding unstained slides with a surgical blade, and miRNAs were isolated as described above. For the serum cohort, RNA was extracted from 1mL of serum using Norgen Plasma/Serum Circulating and Exosomal RNA Purification Kit (Slurry Format) (42800).

### Next-generation sequencing

High-integrity RNA was used to perform small-RNA library prepartion with NEXTflex™ Small RNA-Seq Kit v2 per manufacturer's instructions. The barcoded library was then bioanalyzed for quality and sequenced on an Illumina Hiseq 2000 at the Rockefeller University's Genomic Resource Center. For analysis, we first performed quality trimming and linker removal (cutadapt v1.8). We then used bowtie2 to align the reads to the human genome miRNA database (hg19). Unmapped reads were then filtered using samtools (v1.3) and resulting miRNA read counts were analyzed for differential expression with the R package DEseq2 (Bioconductor).

### Quantitative reverse-transcription PCR (qRT-PCR)

Reverse transcription was performed using the Thermofisher TaqMan Reverse Transcription kit (4366597). Subsequent quantitative PCR was performed using the Thermofisher Taqman hsa-miR-203 assay (00050) with hsa-miR-331 assay (Thermofisher A25576) as an endogenous control to derive ΔCt values (changes in threshold cycle). Relative miRNA-203 expression levels were derived using 2^^^(−ΔΔCt) method, where ΔΔCt was calculated by subtracting the average ΔCt value in long-term survivors from individual ΔCt values for each sample. MiR-331 was used as a control for all samples given that miR-331 was found to not be significantly different between two survival groups on the initial high-throughput small-RNA sequencing.

### Statistical analysis

Continuous variables were presented using the median, whereas categorical variables were presented using frequency and percentage. Wilcoxon rank sum test and Fisher's exact test were used to compare continuous and categorical variables, respectively, between long-term and short-term survivors. Univariate and multivariate logistic regressions were performed with long-term survivorship as the outcome. *P*-values < 0.05 were considered significant, and were derived from two-sided tests unless otherwise noted. All statistical analyses were performed using R version 3.2.2 (cran.r-project.org) and scatter plots were generated using GraphPad Prism V6.0 (GraphPad Software, CA).
